# Medical students’ personal experiences, religion, and spirituality explain their (dis)comfort with a patient’s religious needs

**DOI:** 10.36834/cmej.69217

**Published:** 2020-08-06

**Authors:** Cindy Schmidt, Joseph Eickmeyer, Meghan Henningsen, Alex Weber, Amanda Pleimann, Seth Koehler

**Affiliations:** 1 Kansas City University of Medicine and Biosciences, Missouri, USA; 2 St. Luke’s Des Peres Hospital, Missouri, USA; 3 St. Luke’s Tri-County Family Medicine, Missouri, USA; 4 Southeast Health Primary Care, Missouri, USA

## Abstract

**Background:**

Physicians often avoid discussing patients’ religious and spiritual concerns, even though most patients (i.e., 50-94%) want integrated care. To address this gap, medical students interviewed a Standardized Patient (SP) who was upset because the daughter did not confront her fiancée about converting to Orthodox Judaism. Students reflected on how their own religion and spirituality affected engaging with their patient.

**Methods:**

With a 97% response rate, 231 first-year medical students responded to open-ended questions about their patient encounter. For this quantitative content analysis, we used inductive reasoning, identifying three themes: (1) impact of students’ own religion on their comfort, (2) change in comfort, and (3) their learning. We used deductive reasoning to compare qualitative results from half of the students who began the curriculum with a questionnaire about their own spirituality with the other students completing afterwards.

**Results:**

Most students said being religious positively influenced their comfort, whether they were also Orthodox Jewish or from a different religion. Among uncomfortable students (6.5%), some attributed this to not being religious. Some students (4.8%) grew more comfortable discussing the religious issue, and 18.2% became uncomfortable due to lacking knowledge of Orthodox Judaism and the awkwardness of the topic. Students who had completed the questionnaire beforehand gave more comments about connecting with their patients than students who completed the questionnaire afterwards (X^2^=11.047, p<.001).

**Conclusions:**

Students’ own religion influenced their comfort with discussing religious concerns, with some feeling more connected and others becoming uncomfortable. This finding helps inform medical educators about teaching mind-body-spirit care.

Résumé

## Abstract (fr)

**Contexte:**

Les médecins évitent souvent de discuter des préoccupations religieuses et spirituelles des patients même si la plupart d’entre eux (soit entre 50 et 94 %) désirent recevoir des soins intégrés. Pour combler cette lacune, les étudiants en médecine ont interviewé un patient simulé (PS) qui était contrarié parce que sa fille n’avait pas confronté son fiancé à l’idée de se convertir au judaïsme orthodoxe. Les étudiants ont réfléchi sur l’impact qu’avait leur propre religion et spiritualité sur la façon dont ils abordent leur patient.

**Méthodes:**

Avec un taux de réponse de 97 pour cent, 231 étudiants de première année en médecine ont répondu à des questions ouvertes sur la rencontre avec leur patient. Dans le cadre de cette analyse de contenu quantitative, nous nous sommes servis du raisonnement inductif et avons déterminé trois thèmes :(1) impact de la religion des étudiants sur leur niveau de confort, (2) changement du niveau de confort et (3) leur apprentissage. Nous avons utilisé le raisonnement déductif pour comparer les résultats qualitatifs obtenus pour la moitié des étudiants qui ont commencé leur cursus en remplissant un questionnaire sur leur propre spiritualité, l’autre moitié ayant répondu au questionnaire subséquemment.

**Résultats:**

La plupart des étudiants ont affirmé que d’être religieux avait eu un impact positif sur leur niveau de confort, qu’ils soient juifs orthodoxes ou d’une religion différente. Parmi les étudiants inconfortables (6,5 %), certains ont attribué ce sentiment au fait qu’ils n’étaient pas religieux. D’autres (4,8 %) se sont sentis plus à l’aise après avoir discuté le problème religieux et 18,2 % d’entre eux se sont sentis moins à l’aise en raison de leur manque de connaissance sur le judaïsme orthodoxe et du sujet embarrassant. Les étudiants qui avaient rempli le questionnaire auparavant ont fourni plus de commentaires sur leur façon d'aborder leurs patients que ceux qui y avaient répondu après (*X^2^*=11,047; p<,001).

**Conclusions:**

La religion des étudiants a eu un impact sur leur degré d’aisance au moment de discuter les questions religieuses; certains se sentant plus proches et d’autres, plus inconfortables. Cette conclusion informe les éducateurs en médecine de l’importance d’enseigner les soins du corps, de l’esprit et de l’âme.

## Introduction

Most seriously ill patients use their religiosity and/or their spirituality to cope with their illness, though results are more mixed about whether it helps comfort them.^1^^-^^4^ Even among patients who are healthy, 94% of patients who rated themselves as religious or spiritual want to talk to their physician about their beliefs, while 50% of those who did not rate themselves as religious or spiritual still want to be asked about it by their physician.^5^

Scholars and theologians debate with great vigor the meaning of the terms “religious” and “spirituality”. As a research team of six, we share the spirit of this debate, though we recognize a need to offer some clarity in their distinction. Without attempting to give a formal or thorough definition, with great respect for the complexity, and in the interest of brevity for this manuscript, we offer this distinction in our own usage of these terms: (1) religion – an organized culture with a set of beliefs and practices that may or may not include spirituality and that usually centers around one or more deities and that often produces meaning-making, (2) religious – an adjective describing an individual who ascribes to some set of beliefs and/or engages in some set of practices relating to one or more religions and that may or may not include spirituality and who may or may not believe in one or more deities, (3) spirituality – connecting with the breath/spirit of one or more deities / life force that generates from an ethereal source, sometimes conceptualized as one or more deities, and both filled by and expressed, in various manners, for which scholars disagree on the type, number, and essence, typically ranging from 3 to 20.^21^ (See Note 1 for further comments.)

There is still a substantial gap between what physicians do and what patients want them to do.^6^ Less than 33% of physicians feel that they should ask about their patient's religion or spirituality, and those most likely to ask are either “religious and spiritual” or they are “spiritual but not religious”.^7^^-^^8^ Ernecoff *et al*. found that 78% of intensive care patients’ decision-makers rated religion and spirituality as important, but only 3% said that a physician addressed their concerns.^9^ Even with efforts over the past 20 years to bridge the gap, the divide persists.

Medical education provides an opportunity to better align patients’ needs and preferences with physician practices by teaching communication skills about how to talk with patients about their religious and spiritual needs. One effort to motivate medical students to discuss patients’ religious and spiritual concerns found it useful to provide behavioral exposure to a patient with religious and spiritual concerns.^10^ Supporting this approach, medical students who are religious and/or spiritually open themselves have greater empathy with their patients and tend to use their religiosity and/or spirituality to bear the emotional impact of their patients’ suffering.^11^^-^^12^ In contrast with the literature indicating low rates of physicians’ addressing their patients’ religious and spiritual concerns, medical students favor experiential curricula intended to support their own personal religious and/or spiritual growth and values.^13^ Medical education needs to teach students how to comfortably and capably integrate their patients’ religion and spirituality into their care, in order to best provide truly patient-centered care.^13^ Our study looked at students’ comfort with discussing their patient’s religious needs as part of their overall care and explored how focusing on their own spirituality may have influenced their ability to hold this discussion. These two components together look at how medical education can teach the importance of providing mind-body-spirit care to patients while also supporting the mind-body-spirit of our students.

To better understand how to teach mind-body-spirit care to students, our study looked at first year (second-semester) medical students’ written reflections regarding their reactions to an experiential curriculum with an SP case about a religious issue. Our exploration looked at factors influencing students’ comfort with having this discussion, which we hope will shed light on the literature indicating low rates of physicians addressing patients’ religious and spiritual concerns. The reflections analyzed here are one component of a larger data set collected for this study.

## Methods

### Study sample

Scheduled in groups of 15, a faculty member (CS) pre-briefed the first year (second semester) allopathic medical students to their SP encounter. Reflection questions are a routine component of this simulation curriculum in the Introduction to Clinical Medicine courses. Of the 237 students invited to participate in the study by providing their reflections to the research team, five students declined, and one student consented but did not provide any responses, for a total of 231 participants. The IRB at American University of the Caribbean School of Medicine (AUC) approved the study, #2015-004, on 2/6/2017.

### Study design

This study was part of a larger study looking at students’ responses to an SP interview about a religious issue. On the first day of the simulation, 111 students met individually with their SP, considered their own spiritual needs and religious beliefs, then responded to the narrative self-reflection questions analyzed in this study.^14^ On the second day, 116 students (plus another who rescheduled later) considered their own spiritual needs and religious beliefs, met with their SP, then responded to the same narrative self-reflection questions. In order to avoid overwhelming students with lengthy questionnaires, we did not include additional measures, such as empathy, for example. To ensure students’ anonymity, we did not collect common requested demographic information, since it could potentially make them identifiable.

### Curriculum

The faculty member who pre-briefed students (CS) reminded them about elements of the social history, then instructed them to conduct a focused social history. The pre-briefing emphasized that students would neither be graded nor observed (by peers or by faculty) in order to minimize performance anxiety. Students met with their SP for 15 minutes, then moved to a room where they were seated at individual computers and wrote responses to reflection questions. See Table 1.

**Table 1:** Student self-reflection questions and *a priori* qualitative codes

Reflection question		*A priori* codes
1	How did you like this simulation activity? Please describe	•Reflections about the simulation experience •Love the unique qualities of it •Want more SP experiences •Want to do this again •Love how great the SPs are •Didn’t like filling out the questions •Liked filling out the questions
2	What have you learned?	•Reflections about learning •Learned a lot •Need to learn a lot •Comfortable/uncomfortable with the feedback from my SP
3	How did your religious/spiritual beliefs impact your comfort with and ability to discuss your patient’s religious concerns?	•Reflections about religion •Comfortable •Uncomfortable •My religion didn’t impact our conversation •My religion impacted our conversation
4	Describe any changes in your comfort when discussing religious concerns with your SP.	
5	Other comments?	

### SP case

Female and male SPs received training for their character who presented with headaches. The patient’s daughter was engaged, and they had conflict whenever discussing the wedding, as the patient wanted the daughter’s fiancée to convert to their faith - Orthodox Judaism. We selected this particular religion for this case because many of our students would be relocating for their third year to do their rotations in a predominantly Orthodox Jewish community, recognizing that there would be a need for additional curricula addressing needs arising from other religious traditions.

Two minutes into the encounter, the patient received a text message from their daughter. The patient grew visibly upset by increasing the pace of their breathing, shifting their weight around, and exerting a frustrated sigh. The patient stated that their daughter had yet again failed to talk with her fiancée about converting to their faith (he was a lapsed Catholic). The patient explained the importance of their faith and why their future son-in-law would need to convert to Orthodox Judaism. The patient further expressed fears that due to the upcoming marriage, the daughter could be rejected by the synagogue if the son-in-law did not convert.

The curriculum design team consulted with an Orthodox rabbi to obtain pertinent social history information to enhance validity of the case. Nineteen SPs were trained to deliver this case. They received information and resources to enrich their understanding of the social complexity of the patient’s religious issue. One trainee expressed personal discomfort with the necessity of the patient to pressure the daughter and so declined to contribute to delivering this curriculum. Eighteen SPs demonstrated they mastered the role to the SP Program Director. Seventeen SPs delivered the case (and one remained on standby), meeting one-on-one with students in multiple sessions over the span of two days.

### Analytic strategy

We approached the qualitative coding by first discussing our philosophical perspective on reality – a critical realistic epistemology, in that we believe truth exists in a real world, though we can only know a construction of reality. Acknowledging the pervasiveness of perceptual bias, we sought to strengthen the rigor of our analyses by using reflexivity (considering and naming our own potential sources of bias); triangulation (using multiple coders); and looking for deviant cases (attempting to disconfirm our findings). For reflexivity, we reflected individually, in pairs, and as a full team. Each team member considered their own religion and spirituality; their beliefs about integrating it in patient care; and their values about teaching it, for example. Team members identified instances where their personal beliefs and experiences differed from, and were similar to, the reflections we were coding, and we discussed how that may or may not have impacted coding and interpretive decisions. Even with these efforts to minimize bias, qualitative research remains influenced by the perspectives of the researchers. For these reasons, we frequently reminded each other that our explorations and findings were unique to the group of students we studied.

CS had previous experience with research in medical education and also with qualitative analysis, so she trained the coders and observer in content theme analysis and led the coding process.^15^^-^^16^ To assist with training the coders, CS generated an initial list of *a priori* codes based on her previous research, a review of literature, and a first look through the data. This starter set of *a priori* codes consisted of three themes which were based on the clear distinctions in content in the first three student self-reflection questions. See Table 1 for the students’ self-reflection questions and set of *a priori* codes. (To compare how these changed once coding and interpretation finished, see Tables 2, 3, and 4.)

**Table 2:** Themes about medical students’ learning experience, with representative student reflections

Theme	Subtheme	Representative quote
Overall perception of the simulation:		
Positive	Less pressure and more one-on-one with patient	*I really liked speaking to the patient one on one without having anyone observing me*
	No checklist	*I thought it was a more helpful exercise in patient interaction than when I had a checklist of questions to ask*
	General	*I liked this simulation more than previous simulations in the past*
Negative	Too much freedom, not enough structure	*I did not like the fact that we were given so much freedom; however, I felt like at times I didn’t know what to ask exactly*
Students’ learning from the simulation:		
Positive	Patient connection and empathy	*I was able to practice being present with the patient and establishing rapport*
	Improved talking	*I learned how to communicate professional[ly] as a future physician*
	Improved listening	*To listen to what patients have to say even if they are not talking about something that directly pertains to their medical care*
Negative	Nothing learned	*I didn’t learn anything*

**Table 3:** Themes about impact of students’ own beliefs and spirituality on comfort with their SP encounter

Impact on Comfort	Theme	Representative quote
Positive		
	Different religion than patient	*As a Muslim, I could understand some of her concerns about kosher meals and proximity to her religious institution*
	Same religion as patient	*We connected over a shared belief, so it enables us to establish good rapport*
	Student did not indicate whether spiritual or religious	*I was able to communicate that I was empathic towards the patient’s situation despite the fact that I was not very familiar with all of the religious practices and cultural beliefs*
	Not spiritual or religious	*It’s not so much religious beliefs as it is just being able to put yourself in someone else’s shoes*
Negative		
	Different religion than patient	*I felt a little distant in the beginning being of different faith than the patient*
	Student lacked knowledge about patient’s religion	*I was not very familiar with all of the religious practices and cultural beliefs/practices*
	Student found religion to be a sensitive topic	*I’m concerned about crossing a line into an area a patient considers off limits*
	Student not spiritual or religious	*I felt like I was not the best person to have that conversation with*

**Table 4:** Changes in students’ comfort from participating in the SP encounter

Nature of change	Impact on comfort, explanation	Representative quote
Positive		
	Increased comfort	*If anything, I became more comfortable*
Neutral		
	No change in comfort	*No changes in comfort. My job is to treat patients and religion is not a factor in that*
Negative		
	Lack of knowledge	*I think I was afraid to show my non-familiarness [sic] about the topic to my patients*
	Religion is a sensitive topic	*I was very uncomfortable during the part when the patient told me about the situation with her daughter. It was the main reason the interview time was so short. I had something to say, but since religion is a sensitive topic, I refrained from commentating [sic] on it and it probably made me come across as disconnected*
	Not spiritual or religions	*My lack of religious/spiritual beliefs actually inhibited my ability to speak about the topic in a comfortable manner with the patient*
	Student a different religion than patient	*I think that because I was not of the same faith as my patient, that I didn’t quite know how to advise her on what to do. The topic generally makes me uncomfortable to discuss with other people, but I see the necessity of it*.

Beginning with a set of *a priori* codes introduces a source of bias. To minimize the effect of this, CS trained the coders (AP, AW, MH, and SK) and observer (JE) to modify the set of codes as much as they wanted; to look intently for deviant cases; and to be open to emergent codes. Once the coding process began, CS refrained from commenting about the students’ reflections or possible themes, as her prior research could have influenced our coding decisions, which could have limited the integrity of our qualitative inquiry.^15^^,^^16^

In the first phase, we coded reflections individually, creating emergent codes to add to the *a priori* codes. We reflected on our coding decisions as pairs, then as a whole group, to use analyst triangulation as a strategy to improve the rigor of our qualitative analysis. Using an iterative process, we revised our coding and negotiated until we reached consensus on applying our final set of themes and subthemes. CS and JE observed all discussions to listen for the possibility of bias influencing our coding decisions. In some of these discussions, coders considered their personal beliefs and values to explore areas of similarity and difference from the reflections they were coding. The final set of coded themes and subthemes varied considerably from the initial set of *a priori* codes.

We used quantitative content analysis looking at the frequency rates of different themes. For themes that we coded once per student (e.g., comfort with discussing the religious issue, same or different religion than patient), we conducted frequency counts and compared them with a chi-square analysis.^17^^,^^18^^,^^19^

## Results

### Participation rate

Ninety-seven percent of students experiencing this curriculum agreed to share their reflections with the research team (*n* = 231).

### Qualitative themes

***Simulation experience and students’ learning*.** Students commented that they liked the simulation experience (*n* = 80, 34.6%), especially focusing on the patient’s story and not a social history checklist (*n* = 90, 40.0%). A few students did not like the simulation (*n* = 4, 1.7%). Most frequently, students stated the simulation improved their conversational skills overall (*n* = 140, 60.6%), their empathic connection with their patients (n=89, 38.5%), and their listening skills (*n* = 31, 13.4%). A few students commented that the simulation was more of a negative experience for them (*n* = 4, 1.7%). See Table 2 for themes, subthemes, and representative quotations.

***Religious impact on students’ comfort*.** Many students described their own religion as positively impacting their comfort with discussing their patient’s religious issue (*n* = 93, 40.6%), and this was often the case for students from a different religious background than the patient (*n* = 61, 26.4%). Some students stated their religion had a negative impact on their comfort (*n* = 15, 6.5%), with a few students indicating they were not religious (*n* = 12, 5.2%). Several students (*n* = 64, 27.7%) said their religion did not impact their comfort or they did not refer to their comfort (*n* = 50, 21.6%). Percentages add up to more than 100% because some students gave more than one type of comment to this self-reflection question. See Table 3 for themes, subthemes, and representative quotations.

***Changes in comfort*.** Most students did not experience any changes in their comfort while discussing their patient’s religious issue (*n* = 154, 66.7%), and a few became more comfortable (*n* = 11, 4.8%). Among the students who became uncomfortable (*n* = 42, 18.2%), their lack of knowledge about religion (*n* = 19/42, 45.2%) and awkwardness/sensitivity of the topic (*n* = 12/42, 28.6%) were their primary reasons (i.e., meaning that students were not sure if they were allowed to discuss religion at all or they did not have a sense of the boundary between being a student doctor and being a chaplain). Percentages add up to less than 100% because we coded some students’ reflections as deviant cases (*n* = 24, 10.4%), as they were unrelated to the self-reflection question and to the other students’ comments. See Table 4 for themes, subthemes, and representative quotations.

### Quantitative content analysis

More students who had considered their own spirituality and religious beliefs before their SP encounter reported comments about feeling empathic toward their patients, and more students who had considered their spirituality and beliefs afterward gave comments about improving their conversation skills (*X^2^* = 11.047, *p* < .001). Comparing the students who considered their spirituality and beliefs beforehand to those who considered their spirituality and beliefs after their SP encounter, students did not differ significantly (i.e., non-significant chi-square tests) in their comfort - whether their religion impacted their comfort, or whether they experienced any changes in their comfort once they began discussing their SP’s religious issue.

## Discussion

Students reflected on their responses to this simulation encounter, an experiential way of learning how to integrate mind-body-spirit care by attending to a patient’s religious issue. Many believed they improved their communication skills, in general, and their empathic connection with their SPs. Close to half of the students felt comfortable with this discussion of religious needs, some grew uncomfortable, and a small minority became more comfortable. Consistent with the literature calling for experiential learning that supports their own spiritual growth, our results similarly indicate that students liked this method of learning.^10^

Students who considered their own spirituality and religious beliefs before their SP encounter made more comments about feeling connected and empathic than the students who considered their spirituality and beliefs after their SP encounter.^14^ Our results are consistent with previous research that found increased empathy for patients among medical students after they completed coursework in empathy and spirituality, as well as results indicating medical students with greater religiosity and spiritual openness also had greater empathy in these cases.^11^^-^^12^^,^^20^ Although we did not find that considering their own spirituality and beliefs helped them feel more comfortable discussing their patient’s religious issue, the act of considering their own spirituality and beliefs seemed to help them get in touch with their own spirituality and humanity and with that of their patient, based on their comments about feeling connected and empathic with their patients. Perhaps this personal consideration beforehand helped them focus less on worrying about doing the social history, instead being more grounded with themselves and the human-to-human experience of the SP encounter, opening them emotionally to feeling more empathic and connected.

Students who were religious said that being religious helped them feel more comfortable discussing the religious issue with their SP, whether they were Orthodox Jewish or from a different religion. Students who were uncomfortable discussing the religious issue identified two reasons for their discomfort: lacking knowledge and finding religion to be a sensitive topic. Were they uncomfortable because they felt uninformed and underprepared; were they uncomfortable because they had a prior negative experience with religious discussions; or were they uncomfortable because they were unsure of the boundaries of what and how to discuss religious issues? Individual reflection comments point to all of these reasons, though we cannot conclude how widely endorsed each may be or whether some students may have held multiple reasons for their discomfort. For educators designing curricula teaching mind-body-spirit care, it may be advisable to anticipate and address these potential aspects of student discomfort. From an instructional design perspective, many students commented that they liked the freedom of not being observed by peers or faculty and not being graded. Although there are compelling benefits to observation with formative and summative feedback, there may also be compelling benefits to reducing the performance aspect of simulation education. Perhaps students felt more courageous to try something new. Consistent with the purpose of qualitative research, our explorations raise these and other interesting possibilities.

## Limitations

Qualitative research necessarily presents a limitation from the unseen biases in the researchers, a natural result of which is that findings may not be generalized. Even though we used quantitative content analysis in our qualitative study, our results represent our students’ reflections on their own unique experience. Another limitation to our study is that some students could have interpreted the reflection questions differently or may have thought about their experiences differently on the second day of the workshop. Along this line, structured qualitative questioning unfortunately constrains the scope of content participants provide. Though not as limited as an objective questionnaire, even specific open-ended questions necessarily limit participants’ responses. A further challenge, beginning with a priori codes may have biased coders’ decisions and / or affected their ability to perceive emergent codes.

The SP case we used represented only one religion, Orthodox Judaism, so we did not learn about students’ reactions to interviewing an SP from a different religion.

As a qualitative study, we did not gather ratings on strength of empathy or change in empathy, as would be found with a standardized measure such as the Jefferson Scale of Empathy.

Some students may not have fully immersed themselves during the SP encounter, and this could have impacted their empathy or comfort. Students with lower self-reflective capacity or a lower willingness to share their reflections may not have expressed fully their reactions.

### Future directions

Future research could explore other micro-experiences that help create similar openness and if that openness then leads to engaging (rather than avoiding) discussions about religious and spiritual needs. Pre-encounter mindfulness or other experiences that emphasize humanism could potentially generate similarly positive feelings of connection and empathy. Do SPs agree that students engage more in a lower stress context?

Developing a curriculum with information about different religious traditions and incorporating reflection and discussion, may address students’ concerns about lacking knowledge about religion. A retrospective pre-curriculum self-assessment could check to see if this knowledge improves students’ competence with discussing religious needs during an SP encounter.^24^

Qualitative research offers the strength of exploring an area that is underdeveloped. An expected outcome is to generate hypotheses that can then be examined in future research. There are many possible research designs that could advance this inquiry. For those wanting to further develop hypotheses, conducting focus group interviews or individual interviews is recommended. For those wanting to examine hypotheses, an experimental or quasi-experimental design is recommended. Another good option would be a mixed-method study combining questionnaires and interviews, for both students and SPs.

## Conclusions

Many students reflected that the SP encounter improved their communication skills and enhanced their empathy. They also indicated that being religious, from any tradition, helped them feel comfortable discussing their patients’ religious issue. Those students who took time to reflect on their own spirituality before their SP encounter reported more comments about feeling empathic and connected with their SP than was reported by students who did not engage in this reflection beforehand. Physicians should become comfortable and routinely address the religious and spiritual needs of patients in order to fully provide patient-centered care. As we learned from our students, however, this may be hindered due to lack of knowledge, prior negative experiences, and lack of clarity around boundaries.

## References

[1] Koenig HG, King DE, Carlson VB Handbook of Religion and Health. 2nd ed Oxford, England: Oxford University Press, 2012.

[2] Alcorn SR, Balboni MJ, Prigerson HG, Reynolds A, Phelps AC, Wright AA, Block SD, Peteet JR, Kachnic LA, Balboni TA “If God wanted me yesterday, I wouldn’t be here today”: religious and spiritual themes in patients’ experiences of advanced cancer. J Palliat Med. 2010;13:581-8. 10.1089/jpm.2009.0343. 20408763

[3] Park CL Spirituality and meaning making in cancer survivorship, in: Markman K, Proulx T, Lindberg M, eds The Psychology of Meaning. Washington, DC: American Psychological Association, 2013:257-77. 10.1037/14040-013

[4] Preau M, Bouhnik AD, Le Corollar Soriano AG Two years after cancer diagnosis, what is the relationship between health-related quality of life, coping strategies and spirituality? Psychol Health Med. 2013;18:275-286. 10.1080/13548506.2012.736622. 23140373

[5] Ehman JW, Ott BB, Short TH, Ciampa RC, Hansen-Flaschen J Do patients want physicians to inquire about their spiritual or religious beliefs if they become gravely ill? Arch Intern Med. 1999;159:1803-6. 10.1001/archinte.159.15.1803 10448785

[6] Best M, Butow P, Olver I Do patients want doctors to talk about spirituality? A systematic literature review. Pat Educ Couns. 2015;98:1320-8. 10.1016/j.pec.2015.04.017. 26032908

[7] Franzen AB Influence of physicians’ beliefs on propensity to include religion/spirituality in patient interactions. J Rel Health. 2018;57:1581-97. 10.1007/s10943-018-0638-7. 29876717

[8] Monroe MH, Bynum D, Susi B, et al Primary care physician preferences regarding spiritual behavior in medical practice. Arch Intern Med. 2003;163:2751-56. 10.1001/archinte.163.22.2751. 14662629

[9] Ernecoff NC, Curlin FA, Buddadhumaruk P, White DB Health care professionals’ responses to religious or spiritual statements by surrogate decision makers during goals-of-care discussions. JAMA Intern Med. 2015;175:1662-9. 10.1001/jamainternmed.2015.4124 26322823

[10] Chibnall JT, Duckro PN Does exposure to issues of spirituality predict medical students’ attitudes toward spirituality in medicine? Acad Med. 2000;75:661 10.1097/00001888-200006000-00020 10875513

[11] Balboni MJ, Bandini J, Mitchell C, et al Religion, spirituality, and the hidden curriculum: Medical student and faculty reflections. J Pain Symptom Manage. 2015;50:507-15. 10.1016/j.jpainsymman.2015.04.020. 26025271 PMC5267318

[12] Damiano RF, DiLalla LF, Lucchetti G, Dorsey JK Empathy in medical students is moderated by openness to spirituality. Teach Learn Med. 2017;29:188-195. 10.1080/10401334.2016.1241714. 27997222

[13] Mitchell CM, Epstein-Peterson ZD, Bandini J, et al Developing a medical school curriculum for psychological, moral, and spiritual wellness: Student and faculty perspectives. J Pain Symptom Manage. 2016;52:727-36. 10.1016/j.jpainsymman.2016.05.018. 27693904 PMC5319601

[14] Fisher J You can’t beat relating with God for spiritual well-being: Comparing a generic version with the original spiritual well-being questionnaire called SHALOM. Religions. 2013;4:325-35. 10.3390/rel4030325

[15] Schmidt CA, Patterson MA, Ellis AM, Nauta HL Religious and spiritual assessment: A Standardized Patient curriculum intervention. Clin Simul Nurs. 2017;13:314-20. 10.1016/j.ecns.2017.05.007.

[16] Schmidt C, Nauta L, Patterson M, Ellis A Medical students’ (dis)comfort with assessing religious and spiritual needs in a Standardized Patient encounter. J Relig Health. 2019;58(1):246-58. 10.1007/s10943-018-0714-z 30306388

[17] Davis C How to quantify qualitative data: What is the significance of your data? 2014 Available at: http://www.qsrinternational.com/nvivo/nvivo-community/blog/how-to-quantify-qualitative-data. [Accessed June 21, 2018].

[18] Loehnert S About statistical analysis of qualitative survey data. International Journal of Quality, Statistics, and Reliability. 2010;Article ID 849043:12 pages. 10.1155/2010/849043.

[19] Social Science Statistics. Chi-square test calculator. 2018 Available at: https://www.socscistatistics.com/tests/chisquare. [Accessed July 3, 2018].

[20] DiLalla LF, Hull SK, Dorsey JK Effect of gender, age, and relevant course work on attitudes toward empathy, patient spirituality, and physician wellness. Teach Learn Med. 2010;16(2):165-170. 10.1207/2153280115tlm1602_8 15276893

[21] Fisher J From the beginning to spiritual well being. Духовність особистості: методологія, теорія іпрактика. 2016;3:74-97.

[22] Streib H, Hood RW Modeling the religious field: Religion, spirituality, mysticism, and related world views. Implicit Religion. 2013;16(2):137-155. 10.1558/imre.v16i2.137

[23] James W The varieties of religious experience: A study in human nature. Abingdon-on-Thames, United Kingdom: Routledge; 2003 10.4324/9780203398470

[24] Zhao R, D’Eon M Five ways to get a grip on grouped self-assessments of competence for program evaluation. Can Med Ed J. 2020 Mar.9 10.36834/cmej.69276 PMC7417831 32821308

